# Solubility of Some Drugs in Aqueous Solutions of Choline Chloride-Based Deep Eutectic Solvent Systems: Experimental Data, Modeling, and the Impact of Solution pH

**DOI:** 10.5812/ijpr-137011

**Published:** 2023-06-28

**Authors:** Samira Zadali Asghar, Raha Kaviani, Ali Shayanfar

**Affiliations:** 1 Biotechnology Research Center, Tabriz University of Medical Sciences, Tabriz, Iran; 2 Student Research Committee, Tabriz University of Medical Sciences, Tabriz, Iran; 3 Pharmaceutical Analysis Research Center, Tabriz University of Medical Sciences, Tabriz, Iran; 4 Editorial Office of Pharmaceutical Sciences Journal, Faculty of Pharmacy, Tabriz University of Medical Sciences, Tabriz, Iran

**Keywords:** Choline Chloride, Deep Eutectic Solvent, Drug, Quantitative Structure-property Relationship, Solubility

## Abstract

**Background:**

The solubility of drugs in water and organic solvents is a crucial factor in numerous pharmaceutical processes. In recent years, a new type of solvent called deep eutectic solvents (DESs) has been developed as a useful solvent for drugs. Choline chloride-glycerol/urea (ChCl-G/U) systems are DESs recognized as a novel category of environmentally friendly solvents. One recent application of this type of DES in water is the solubilization of drugs.

**Objectives:**

This study aimed to investigate the solubility of certain drugs in ChCl-G/U. In addition, the solubilization mechanisms of the DESs studied, and quantitative structure-property relationship (QSPR) models for solubilization were proposed.

**Methods:**

The solubility of 13 drugs in an aqueous solution of the ChCl-G/U system was investigated using the shake flask method. The study was conducted at 10% and 50% mass fractions of the studied systems. Multiple linear regression models were used to develop mathematical relationships between the solubilization of the studied compounds in the presence of ChCl-G/U + water mixture using QSPR models.

**Results:**

The solubility of the compounds showed a significant increase upon adding ChCl-G/U to the aqueous solutions. Based on the data obtained, QSPR models were developed using solubilization ratio and structural descriptors.

**Conclusions:**

The experimental data demonstrates the potential of utilizing ChCl-G/U as a medium to enhance the solubility of poorly soluble drugs in water. Solubilization of solutes in ChCl-G/U + water mixtures could be correlated with the structural properties of drugs. Moreover, the final pH of the solutions in ChCl-U is a critical factor that must be considered when using this system for solubilization.

## 1. Background

Solubility is an important physicochemical property in drug discovery and development stages. More than 75% and 40% of drug candidates and marketed drugs, respectively, have low aqueous solubility. Various techniques for improving solubility, from processability to formulation standpoints, have been reported in the literature. The classic method for solubilization is cosolvency or solvent mixing (1, 2).

Using organic solvents has some disadvantages, for example, high cost, toxicity, causing air pollution, and some adverse effects. For these important reasons, new classes of solvents named deep eutectic solvents (DESs) have been introduced. They are new analogs of ionic liquids formed by mixing two or more pure compounds whose eutectic point temperature is below that of an ideal liquid mixture because of negative deviation from ideality (3-5). One common type of DES is composed of quaternary ammonium salt (e.g., choline chloride) with a hydrogen bond donor (e.g., glycerol and urea) in various molar ratios used for solubility improvement. Compared to classic organic solvents and ionic liquids, DESs derived from choline chloride offer several advantages, including low cost, ease of preparation, biodegradability, biocompatibility, non-toxicity, and chemical inertness with water. Therefore, they present interesting issues from the perspective of green chemistry (6-8).

Recently, these compounds have been applied in various aspects of drug discovery and development, such as serving as a solubilizing agent, facilitating dermal and transdermal drug delivery, extracting from biological matrices, and acting as a therapeutic compound (9-12). Also, DESs are a new generation of solvents, and limited data have been reported about their effects and mechanism on the physicochemical properties of pharmaceuticals. Therefore, the solubility of chemical and pharmaceutical compounds could have a considerable role in applying them to various processes. Modeling the structural parameters can aid in developing quantitative structure-property relationship (QSPR) models, which can help in comprehending the mechanism of solubilization by DES in aqueous solutions.

Common DESs applied in pharmaceutical sciences are mixtures of choline chloride (ChCl) and glycerol (G)/urea (U). They are acceptable pharmaceutical DESs from the toxicity viewpoint and could be named good candidate solvents in green chemistry attitude (13-15). Despite the instability of this type of DES in a binary mixture with water (> 30%) (16), previous studies have shown their capacity for the solubilization of drugs (17). In these systems, urea as hydrotrope (18) and glycerol (19) as cosolvent was proposed as a common mechanism for the solubilization of drugs.

The cosolvent percentage should generally be kept at a minimum concentration to address economic and toxicity concerns. Concentrations of 10% and 50% are commonly used in pharmaceutical formulations (20). These amounts have been chosen to assess the solubilization capacity of the DESs studied in aqueous solutions. In addition, some studies have reported a significant increase in solubility when using DESs. However, there has been a lack of systematic research and investigation into the mechanisms of solubilization by these solvents. Modeling and investigating changes in the pH of the solution can help understand the solubilization mechanism by DESs.

## 2. Objectives

This study investigates the solubility of 13 drugs with diverse structures and physicochemical properties in the aqueous solution of choline chloride and urea or glycerol system. In addition, QSPR models were developed to establish a relationship between increasing solubility and structural parameters to understand the solubilization mechanism and make predictions.

## 3. Methods

### 3.1. Materials

Active pharmaceutical ingredients of studied medicines, i.e., atenolol (Daroupakhsh, Iran), benzoic acid (Merck, Iran), carbamazepine (Arastoo, Iran), carvedilol (Salehan Shimi, Iran), Ibuprofen (Daana, Iran), ketoconazole (Arastoo, Iran), lamotrigine (Arastoo, Iran), phenothiazine (Merck, Germany), phenytoin (Alhavi, Iran), piroxicam (Zahravi, Iran) salicylic acid (Merck, Germany), sulfamethoxazole (Merck, Germany), and tadalafil (Osveh, Iran), were provided from pharmaceutical and chemical companies (Purity: > 99%). Ethanol (96% w/w) was purchased from Jahan Alcohol (Iran), and choline chloride (> 99%), glycerol (> 99%), and urea (> 99%) from Merck (Germany). Lab-made double distilled water was used for the preparation of solutions.

### 3.2. Preparation of Deep Eutectic Solvents Based on Choline Chloride

A DES composed of ChCl + G/U was selected for this study. It was prepared based on a previously reported method in the literature (13, 14). First, the components of DESs, including ChCl as a hydrogen bond acceptor (HBA) and G/U as a hydrogen bond donor (HBD), were weighed using an analytical balance (ATX224, Shimadzu, Japan) with 1×10^-4^ g precision at a specific mole fraction of 1:2, respectively. The measured amounts of these compositions were added into a beaker and sealed firmly with Parafilm. Then, it was heated at 70°C while stirring at 550 rpm for 60 minutes until a clear and uniform liquid was formed. At the end of the synthesis process of DES, the prepared liquids were cooled and used as a new class of cosolvents for drug solubilization.

### 3.3. Solubility Measurement

Many different techniques of solubility measurements have been reported in the literature. The shake flask is a popular and convenient method for solubility determination (21), which was applied in this study. First, solvent mixtures were made by weighing 10:90% and 50:50% mass fractions of DES: water, respectively. Excess amounts of poorly soluble drugs were added into glass vials containing 4 g prepared solvent mixtures. All of the vials were shaken on a shaker (Heidolph, Schwabach, Germany) and placed in an incubator (Kimia Idea Pardaz Azarbaijan (KIPA) co., Tabriz, Iran) at 37 ± 1°C and 180 rpm for 48 hours. According to preliminary studies, all the solutions would reach equilibration over 48 h. The saturated solutions were centrifuged at 6000 rpm for 15 minutes (Tomy Micro One, Tokyo, Japan) and filtered in some cases (Filter Bioscience Membrane Technology Co., 0.22 µm). Afterward, the obtained clear saturated solutions were diluted with ethanol 96% w/w to maintain the absorbance of solutions in the linear range and analyzed by a UV spectrophotometer (Analytik Jena AG, Germany) at the specific wavelength (λ_m_) of each drug (Table 1). Finally, the absorbance of each solution was converted to concentration using the calibration curves, taking into account the dilution factor. The solubility of each drug was then calculated. Details of the calibration curve λ_m_ for concentration determinations are presented in Table 1. Solubilities were determined three times in all cases, and the mean ± standard deviation of data was reported. Solubilities were reproducible within ± 10 %.

**Table 1.** Calibration Curve Details of Studied Drugs

Drug	Calibration Curve Equation ^a^	Linear Range (mg/L)	Lambda (nm)
**Atenolol**	Abs = 0.0063 C – 0.1584	50 - 333.33	328
**Benzoic acid ** ^**b**^	Abs = 0.0521 C + 0.027	10 - 50	225
**Carbamazepine ** ^**b**^	Abs = 0.0456 C + 0.0663	3.125 - 50	280
**Carvedilol ** ^**b**^	Abs = 0.01 C + 0.0063	6.25 - 100	285
**Ibuprofen ** ^**b**^	Abs = 0.0288 C + 0.1642	6.25 - 100	224
**Ketoconazole**	Absa = 0.0032 C – 0.0512	50 - 666.66	291
**Lamotrigine ** ^**b**^	Abs = 0.022 C - 0.0367	5 - 80	302
**Phenothiazine ** ^**b**^	Abs = 0.1398 C + 0.0225	1.25 - 20	253
**Phenytoin ** ^**b**^	Abs = 0.0335 C + 0.0382	3.125 - 50	220
**Piroxicam**	Abs = 0.057 C - 0.0121	3.125 - 50	358
**Salicylic acid ** ^**b**^	Abs = 0.0162 C - 0.0025	12.5 - 200	308
**Sulfamethoxazole**	Abs = 0.1108 C – 0.1216	5 - 16.66	270
**Tadalafil ** ^**b**^	Abs = 0.022 C - 0.0017	3.125 - 50	280

^a^ R^2 ^> 0.99.

^b^ Reported in Ref. (2)

### 3.4. Computational Models and Mechanistic Interpretation of Solubilization in ChCl-G/U+ Water Mixtures

The QSPR models were applied by multiple linear regression models to develop mathematical relations between the solubilization of the studied compounds in the presence of a ChCl-G/U + water mixture. Structural and physicochemical parameters of the solute, including logarithms of the partition coefficient (log P), molecular weight (MW), topological polar surface area (TPSA), Abraham solvation parameters, and melting point (MP), were calculated by ACD-ilab (https://ilab.acdlabs.com/), except for MP which was collected from ChemIDplus (https://chem.nlm.nih.gov/chemidplus). The QSPR models were developed by regression analysis using SPSS 17. Based on the number of compounds (10 < N < 14), a maximum of two descriptors (22 in total) were included in each model. The selection of descriptors was based on their probability values. Furthermore, a mechanistic interpretation of solubilization in ChCl-G/U + water mixture was discussed based on the developed QSPR models, considering the type of solute studied (acidic, basic, or zwitterion) and the percentage of ionization.

## 4. Results and Discussion

The studied drugs, physicochemical and structural parameters, and type of solute (acid, base, neutral, and zwitterion compounds) are shown in Table 2. The solubility of studied drugs in water and the aqueous solution of 10% and 50% of ChCl-G/U+ water is presented in Table 3.

**Table 2.** The Studied Drugs, Physicochemical and Structural Parameters, pK_a_, and Type of Solute (Acid, Base, Neutral, and Zwitterion Compounds)

Solute	log P	Mw	E	S	A	B	V	TPSA	MP(ºC)	Type of Compound
**Atenolol**	0.10	266.3	1.48	1.97	0.78	1.85	2.18	84.6	105	Base
**Benzoic acid**	2.06	138.1	0.75	1.08	0.57	0.44	0.93	37.3	122	Acid
**Carbamazepine**	2.67	236.3	2.12	2.06	0.39	0.92	1.81	46.8	190	Neutral ^a^
**Carvedilol**	4.11	406.5	3.08	3.00	0.62	2.09	3.1	78.4	114.5	Base
**Ibuprofen**	3.72	206.3	0.78	1.01	0.57	0.51	1.78	40.8	76	Acid
**Ketoconazole**	3.55	531.4	3.14	3.76	0.00	2.22	3.72	57.8	146	Base
**Lamotrigine**	-0.19	256.1	2.4	2.13	0.45	0.93	1.65	89.0	217	Base
**Phenothiazine**	4.15	199.3	1.95	1.53	0.13	0.5	1.48	37.3	187.5	Base
**Phenytoin**	2.52	252.3	1.94	2.04	0.44	1.14	1.87	58.2	286	Acid
**Piroxicam**	1.71	331.0	2.56	3.12	0.72	2.12	2.25	104.1	199	Zwitterions
**Salicylic acid**	1.86	122.1	0.91	1.1	0.70	0.4	0.99	68.2	158	Acid
**Sulfamethoxazole**	0.56	253.3	1.99	2.43	0.59	1.21	1.72	102.8	167	Zwitterions
**Tadalafil**	1.43	389.4	3.39	3.27	0.31	2.27	2.7	71.1	302	Neutral ^a^

Abbreviations: log P, partition coefficient; Mw, molecular weight; E, excess molar refraction; S, polarity/polarizability descriptors of the solute; A, the solute hydrogen-bond acidity; B, the solute hydrogen-bond basicity; V, McGowan volume; TPSA, topological polar surface area; MP, melting point.

^a^ Extremely weak basic (essentially neutral).

**Table 3.** Solubility (g/L) of Studied Drugs in Aqueous Solutions of 10% and 50% of DES and Solubility Ratio in DES50% to DES10%

Solute	Water	Water + ChCl-G10%	Water+ ChCl-G50%	Water + ChCl-U10%	Water+ ChCl-U50%	S_ChCl-G50%_/ S_ChCl-G10%_	S_ChCl-U50%_/ S_ChCl-U10%_
**Atenolol**	21.837 ± 1.9653	28.747 ± 1.256	29.483 ± 1.570	31.359 ± 1.118	32.927 ± 1.561	1.03	1.05
**Benzoic acid**	5.859 ± 0.4277 ^a^	9.53 ± 0.511	16.814 ± 1.202	11.159 ± 0.588	35.269 ± 2.456	1.76	3.16
**Carbamazepine**	0.296 ± 0.0139 ^a^	0.32 ± 0.014	2.730 ± 0.230	0.463 ± 0.010	3.930 ± 0.344	8.54	8.48
**Carvedilol**	0.032 ± 0.0030 ^a^	0.159 ± 0.012	0.873 ± 0.027	0.091 ± 0.007	0.620 ± 0.020	5.48	6.79
**Ibuprofen**	0.104 ± 0.0035 ^a^	0.141 ± 0.008	0.242 ± 0.004	1.299 ± 0.263	3.892 ± 0.622	1.72	3.00
**Ketoconazole**	0.008 ± 0.0008 ^a^	0.035 ± 0.001	0.077 ± 0.007	0.028 ± 0.001	0.062 ± 0.006	2.16	2.24
**Lamotrigine**	0.342 ± 0.0161 ^a^	0.506 ± 0.017	2.285 ± 0.018	0.482 ± 0.015	2.126 ± 0.016	4.52	4.41
**Phenothiazine**	0.003 ± 0.0004 ^a^	0.005 ± 0.000	0.099 ± 0.010	0.023 ± 0.0003	0.135 ± 0.015	18.06	5.83
**Phenytoin**	0.056 ± 0.0053 ^a^	0.060 ± 0.005	0.255 ± 0.020	0.104 ± 0.008	0.753 ± 0.060	4.24	7.25
**Piroxicam**	0.032 ± 0.0014 ^a^	0.032 ± 0.002	0.040 ± 0.001	0.571 ± 0.035	2.071 ± 0.036	1.28	3.63
**Salicylic acid**	2.755 ± 0.3031 ^a^	7.056 ± 0.422	12.728 ± 0.265	9.841 ± 0.555	32.386 ± 0.671	1.80	3.29
**Sulfamethoxazole**	0.110 ± 0.0091	0.147 ± 0.009	0.378 ± 0.013	0.489 ± 0.026	2.019 ± 0.063	2.57	4.13
**Tadalafil**	0.016 ± 0.0019 ^a^	0.017 ± 0.002	0.091 ± 0.006	0.018 ± 0.002	0.151 ± 0.010	5.25	8.42

^a^ Reported in Ref. (2)

The aqueous solution of ChCl-G/U_50%_ compared with ChCl-G/U_10%_ has a more significant effect on the solubilization of studied compounds. However, the ratio of solubility in 50% to 10% showed a wide range (1.03 (atenolol) to 18.06 (phenothiazine) in ChCl-G and 1.05 (atenolol) to 8.48 (carbamazepine) in ChCl-U), and the physicochemical and structural parameters give an acceptable correlation between these parameters.

The solubilization ratio in 50% of S_ChCl-G_ to 10% of _ChCl-G_ has an inverse correlation with A (R^2^ > 0.7) as an indicator of the hydrogen bond donor of a molecule after excluding ketoconazole (A = 0). Glycerol is a fully hydrogen-bonded compound in which each molecule participates as a donor in exactly three hydrogen bonds. It shows that the solubilization ratio is relatively less in 50% of ChCl-G + water than in the molecules with high values of A. Different parameters can affect the solubility of solute, i.e., interactions between solute, component 1 (water), component 2 (G), and component 3 (ChCl). Therefore, exact mechanistic solubilization interpretation based on one parameter, e.g., A, is impossible. However, the drugs with more hydrogen bond donor functional groups in higher concentrations of ChCl-G have less solubility improvement.

For the ChCl-U system, the following QSPR model was obtained for the solubilization ratio in 50% of ChCl-U to 10% of ChCl-U:

Log (S_ChCl-U50%_ / S_ChCl-U10%_) = -0.071 + 0.003 × MP + 0.085 × Log P

N = 13, R^2^ = 0.621, SEE = 0.173, F = 8.2, P < 0.05.

Where N is the number of compounds, R^2^ is the coefficient of determination, and F values and corresponding P-values are acceptable statistically in QSPR studies (22). Also, MP has the best parameters for the solubilization by ChCl-U_50%_ compared with ChCl-U_10%_ (R^2^ > 0.4). However, the QSPR model composed of two parameters, i.e., log P and MP, has been given acceptable R^2^ > 0.6. Besides, ChCl-U has a strong effect on the solubility of tadalafil and phenytoin in ChCl-U_50%_ compared with ChCl-U_50%_, which has a high MP value (302°C and 286°C, respectively). Previous studies showed that the MP of molecules is a valuable tool for solubility estimation in water (23) and octanol (24). Generally, MP and logP positively correlate with log (S_50%_/S_10%_). A molecule with a hydrophobic structure and high MP value has more solubilization effect in higher concentrations of the studied system.

**Figure 1.**
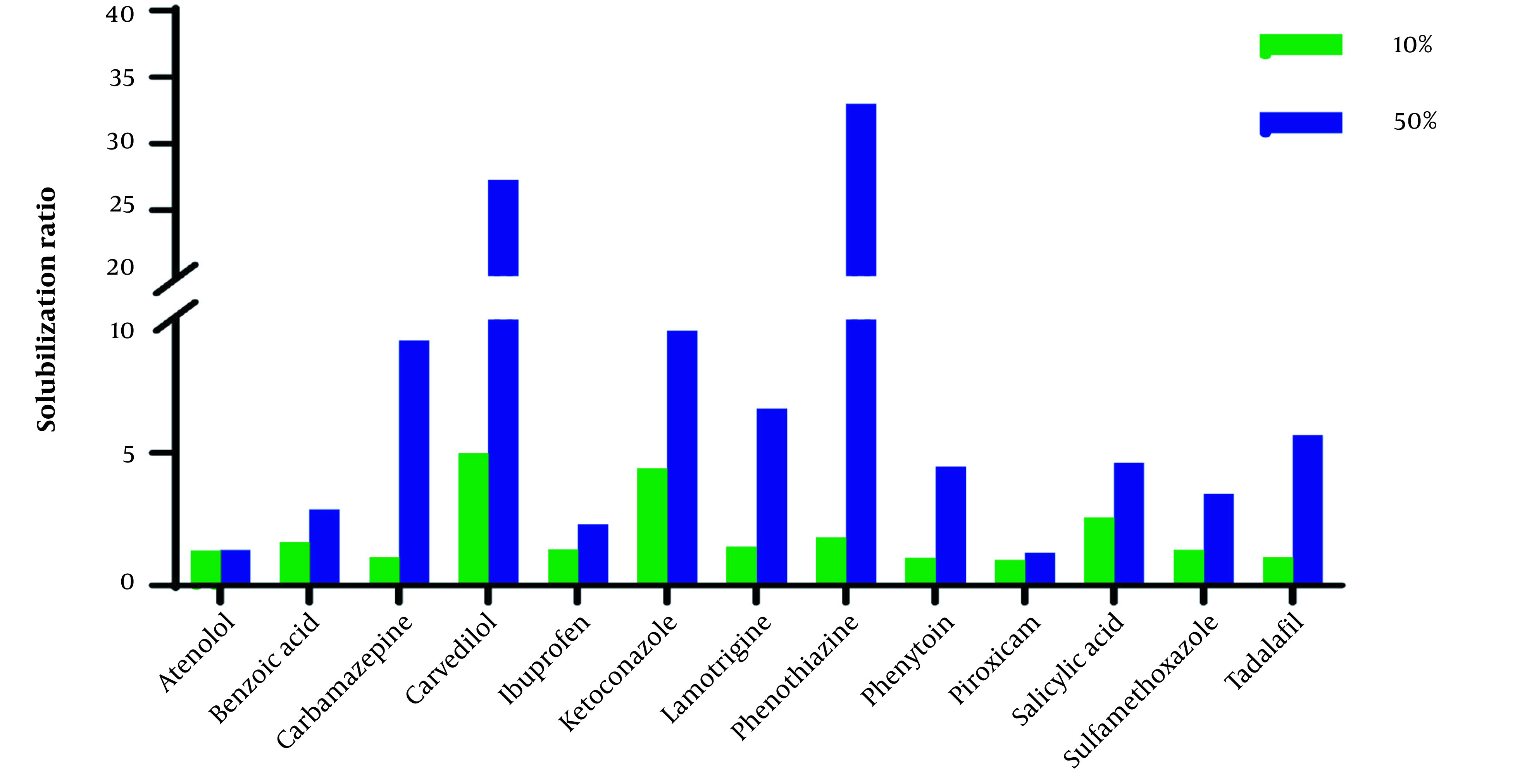
Solubilization ratio of studied drugs in aqueous solutions of 10% and 50% of choline chloride + glycerol

**Figure 2.**
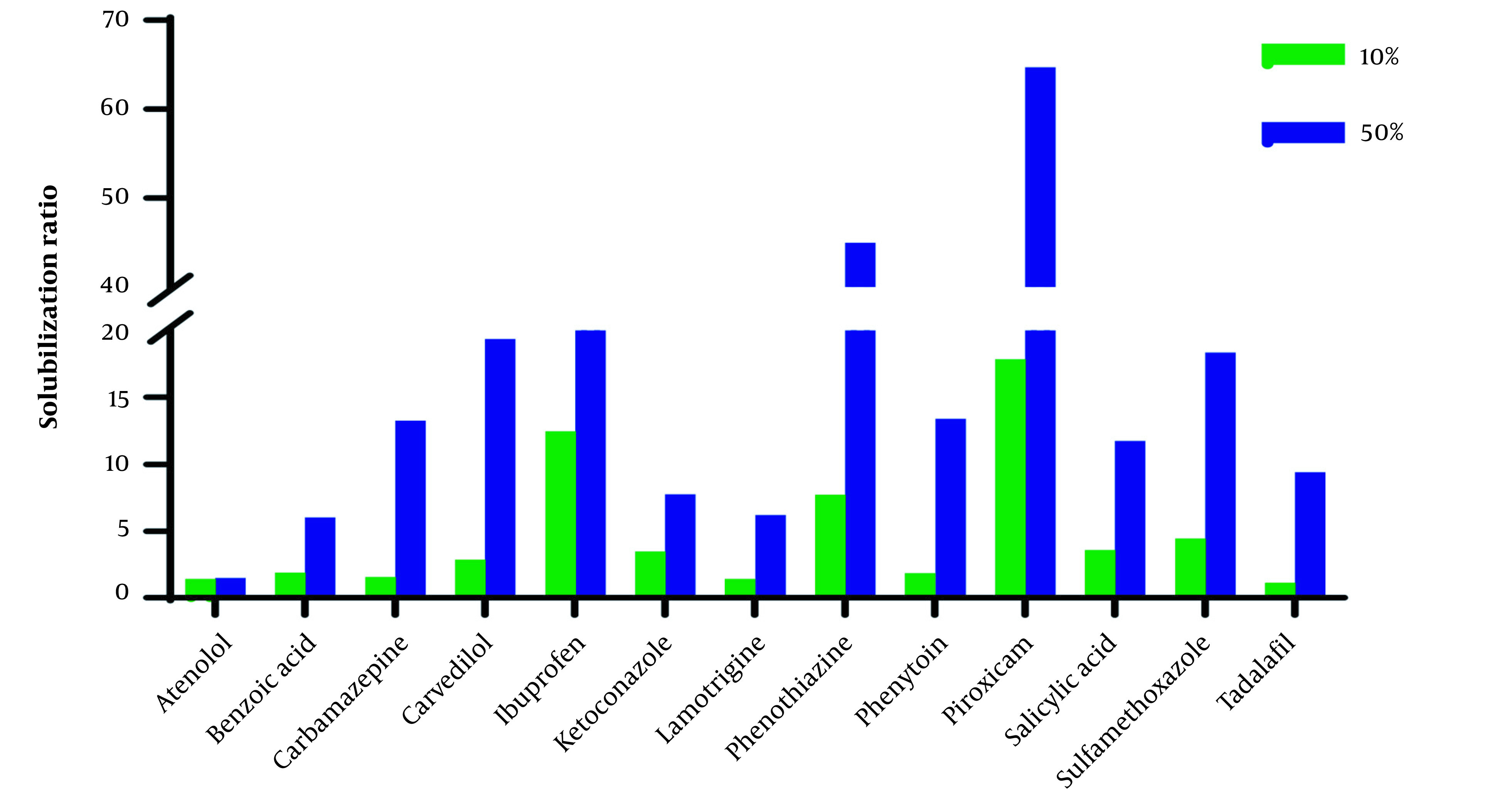
Solubilization ratio of studied drugs in aqueous solutions of 10% and 50% of choline chloride + urea

The solubilization ratio (solubility in ChCl-G/ChCl-U_10%_ and ChCl-G/ChCl-U_50%_ to solubility in water (S_w_)) in Figures 1 and 2 shows that the effect of ChCl-G/ChCl-U on the studied drugs is in a wide range.

The QSPR models for solubilization by ChCl-G are:

Log (S_ChCl-G10%_ / S_w_) = 0.352 + 0.151 × E - 0.003 × MP

N = 13, R^2^ = 0.455, F = 4.2, P < 0.05.

Log (S_ChCl-G50%_ / S_w_) = 0.241 + 0.648 × E - 6.57 × B

N = 13, R^2^ = 0.605, F = 7.5, P < 0.01.

Where E is excess molar refraction composed of molar refraction and volume of a molecule that is calculated by atomic fragmental and the number of bonds in the molecule (25). This parameter has been used for estimating the aqueous solubility of pharmaceuticals in a previous study (26). Also, MP and B are melting point and hydrogen bond basicity, respectively. The obtained models have been proposed for 13 drugs with diverse structures, and they are statistically significant and have acceptable correlations.

The relationship between solubilization ratios in ChCl-U_10%_ of the studied solutes was investigated, and no acceptable model was obtained (R^2^ = 0.172, F = 1.04, P > 0.05). One application of modeling is detecting outlier data. Excluding two solutes, i.e., piroxicam and sulfamethoxazole, give an acceptable model as follows:

Log (_SChCl-U10%_ / S_w_) = 0.361 + 0.161 × log P -0.154 × E

N=11, R^2^=0.680, F=8.5, P < 0.01.

Similar patterns (R^2^ = 0.305, F =2.20, P > 0.05) have been observed in the modeling of solubilization ratios in ChCl-U_50%_, and after excluding piroxicam and sulfamethoxazole, the following model was obtained:

Log (S_ChCl-U50% _/ S_w_) = 0.759 + 0.209 × log P - 0.176 × B

N = 11, R^2^ = 0.740, F = 11.4, P < 0.01.

These models confirm the effective role of logP in the modeling of solubility data in ChCl-U + water mixtures. Comparable results have been reported for solubility prediction of drugs in water (27), water + cosolvent (28), and organic solvents (24). Moreover, Abraham solvation parameters, in agreement with reported studies by Abraham and coworkers and other researchers, have a significant position in the solubility prediction of chemical and pharmaceutical compounds (28-30).

As shown in Table 3, the outlier compounds, piroxicam, and sulfamethoxazole, exhibit a significant increase in solubility when 10% and 50% of ChCl-U are present. These compounds do not correlate with structural parameters, particularly logP, and are identified as zwitterions. It could be related to a change in the pH of the solvent. The pH of the dissolution medium significantly changes after the solute is saturated, compared to other compounds. The pH has no significant effect on the solubility of neutral compounds. Acidic and basic compounds can change the pH of the dissolution medium in water. As known, ChCl-U solutions of 10% and 50% have pH values of 7.5 and 8.3, respectively. The pH values of the final solution in water, ChCl-U_10%_, and ChCl-U_50%_ for all studied basic and acidic compounds were higher and lower than their pK_a_, respectively. Therefore, basic compounds are mostly in non-ionized form, while acidic compounds are in the ionized form (> 50%) based on the Henderson Hasselbalch equation (31, 32). The increased solubilization of acidic compounds in ChCl-U50% may be attributed to ionization. For instance, the saturated solution of ibuprofen (pK_a_ = 4.4) in water showed final pH values of 4.5, 4.9, and 6.4 for ChCl-U10%, ChCl-U50%, and water, respectively. Notably, a significant improvement in the solubilization ratio was observed in ChCl-U50% (Figure 1).

Zwitterion compounds have a v-shaped solubility-pH profile and minimum solubility in neutral pHs (33). The pH values of saturated solutions in water, ChCl-U_10%_, and ChCl-U_50%_ for piroxicam were 5.6, 6.1, and 6.2, and for sulfamethoxazole were 6, 6.4, and 6.7, respectively. The solubility profile of piroxicam in various pHs has been reported in the literature (34, 35). A slight change in pH due to the basic nature of ChCl-U (35), in contrast to ChCl-G (36), which is a neutral compound, can alter the ionization of piroxicam, potentially leading to a significant impact on its solubility. A similar pattern may be correct for sulfamethoxazole. These results confirm the obtained values for solubilization in ChCl-U for the studied zwitterion compounds and act as outliers based on the QSPR models.

Previous studies about the solubility of pharmaceuticals in DES + water mixtures have ignored the role of solution pH. Similar results have been reported in a study on cefixime trihydrate solubility in aqueous solutions of DES (choline chloride and glycolic acid) (37). Glycolic acid also is a relatively potent acidic compound (pK_a_ = 3.6) (14), and the studied DES can change the pH of the aqueous dissolution medium (36). Cefixime is a zwitterion, and its maximum solubility is observed in strongly acidic and basic media because of ionization. Therefore, glycolic acid can convert the studied solute to its ionized form, and solubility enhancement results in the ionization of the amine functional group. A classic inorganic/organic acidic compound can also significantly enhance the solubility of cefixime because of its ionization. In high concentrations of glycolic acid, the medium is acidic, and solubility is significantly increased. Therefore, a considerable rise in solubility in the studied solvent mixture could be related to changes in pH and the solubilization effects of DES (38).

Structural parameters of a solute have an important role in solubilization by DES. Conversely, part of the mechanism for solubilization by the DES aqueous system could be related to its component, i.e., ChCl-U, especially for zwitterion compounds, which should be considered in evaluating the solubility of pharmaceuticals in DES + water mixtures.

## 5. Conclusions

The results indicate the possibility of using DES systems in water to solubilize poorly water-soluble substances. The solubilization ratio in the studied systems could be correlated with the structural parameters of drugs, such as hydrophobicity, hydrogen binding parameters, and solute volume. In addition, the solution's final pH based on acidic or basic components of studied DES is an important factor for evaluating solubilization that should be considered in similar studies. In other words, part of the mechanism for the solubilization of studied DESs could be related to their components which change the pH of the solution and the percentage of solute ionization.

## Data Availability

The data supporting this study's findings are available on request from the corresponding author.
